# Effective Health Communication to Reduce Avoidable Readmission: Enhancing Understanding for Patients and Families

**DOI:** 10.1002/nop2.70187

**Published:** 2025-03-14

**Authors:** Elisabeth Coyne, Karin B. Dieperink

**Affiliations:** ^1^ Griffith University Brisbane Australia; ^2^ Family Focused Healthcare Research Center (FaCe), Department of Clinical Research University of Southern Denmark Odense Denmark; ^3^ Research Unit of Oncology, Odense University Hospital Odense Denmark

Globally, 5% of patients are readmitted within 30 days, costing $17 billion USD, with 27% of these readmissions avoidable with better understanding and follow‐up (Chua and Johnson 2022). Improved communication is central to addressing preventable readmissions, which are costly and often avoidable with effective discharge practices. Communication of health information and follow‐up communication are fundamental to helping patients and families manage at home. Avoidable readmissions are linked to poor long‐term health outcomes and reduced chronic disease management (Xiao et al. 2024).

Good communication in healthcare is more than transferring information to another person; it is about ensuring they truly understand and can use this information (Moore et al. 2024). Communication plays a key role in helping patients and families understand health conditions, solve problems and make informed decisions (Coyne et al. 2025). In this editorial, I will explore communication and strategies to improve communication with patients and families. The relationship between health literacy and communication and the influence of health literacy on patient and family outcomes will be discussed. Finally, recommendations for improving communication between health professionals and families will be presented.

As health professionals, we communicate all the time, but we must ask ourselves, ‘Are we communicating effectively or just talking?’ Communication should be personalised, tailoring the way we speak based on the receiver's responses (Østervang et al. 2021). Tailored communication means there is a shared goal of understanding, to effectively convey the concept to the other person, meeting their communication and cultural needs (Jang et al. 2022). When we reflect on our communication, we must consider whether we are truly sharing information for understanding or just providing facts. Simply talking involves delivering information without adjusting for the recipient's needs, while true communication means ensuring both parties understand the information being shared.

There are various communication styles, and everyone has their own approach to sharing information. It is important to consider both your own communication style and the preferred style of the person you are communicating with. Recognising body language, time spent in the communication interaction, and providing feedback or clarification influence how the other person processes and responds to the information. Engaging with the person by listening attentively, looking for acknowledgment, and simplifying the information are key steps to improving communication (Bishaw et al. 2024).

The first step in overcoming communication challenges is to recognise the audience you are communicating with. Reflecting on how the other person understands the information involves looking for signs of confusion or engagement. In a sample of interviewed nurses, many said they look for facial expressions to check if patients understand, but culturally, many people nod or show understanding even if they are confused (Hogan et al. 2024). Research shows that health professionals often overestimate patient understanding, relying on instinct rather than objective assessment (Hogan et al. 2023).

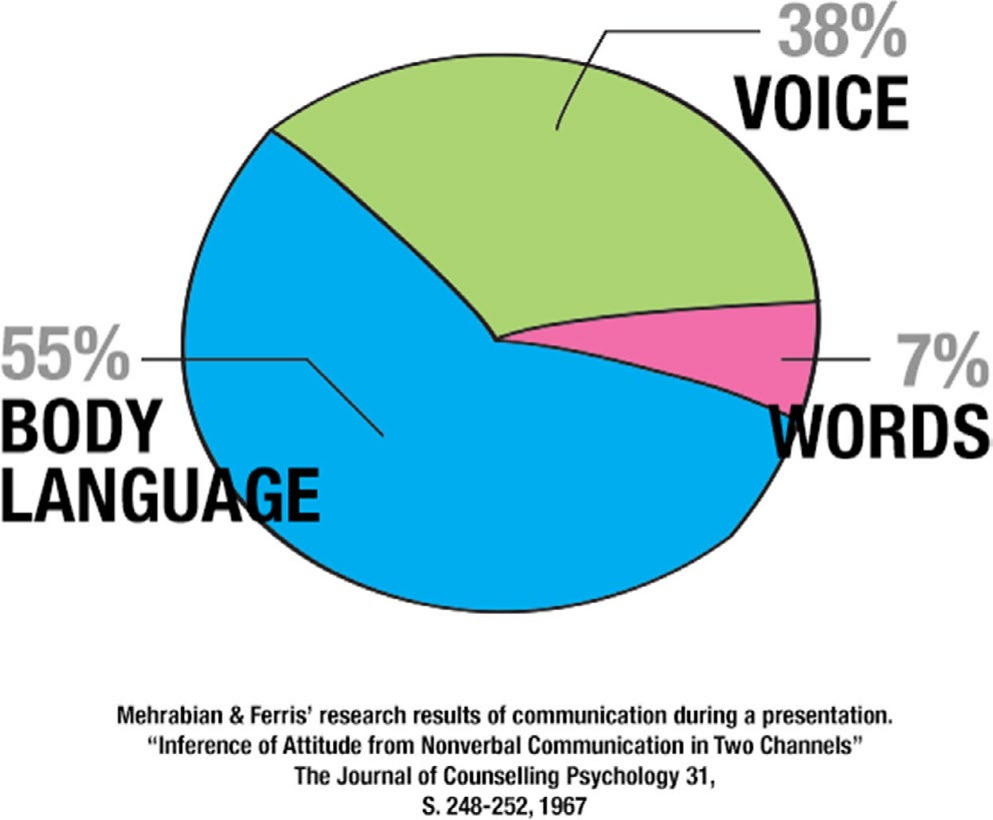

## 1 Health Literacy

Understanding literacy levels is essential for health professionals to gauge the person's ability to understand information. For example, 44% of adults have a literacy level equivalent to primary school, and 38% to senior level; this means health information needs to be broken down into simpler, more understandable sentences (World Health Organization 2023). These challenges are even greater for people from rural, remote, or culturally diverse backgrounds (Aljassim and Ostini 2020; Kozhimannil and Henning‐Smith 2021).

Improving communication between health professionals and families starts with understanding health literacy. Health literacy is defined as the ability to access health services, understand and use health information, and make informed decisions (World Health Organization 2023). Health literacy levels are often lower than expected, with over half of the population struggling to understand medication labels effectively (World Health Organization 2023). For example, many people misunderstand the words salt and sodium or take medication instructions like ‘with meals’ too literally, skipping doses if they do not eat. In a study of rural cancer patients, one participant said, ‘I had no idea what radiotherapy was; I was just shell‐shocked when I found out I was going to get radiation every day’ (Coyne et al. 2019). This highlights the need to clarify information and understand how families share the information.

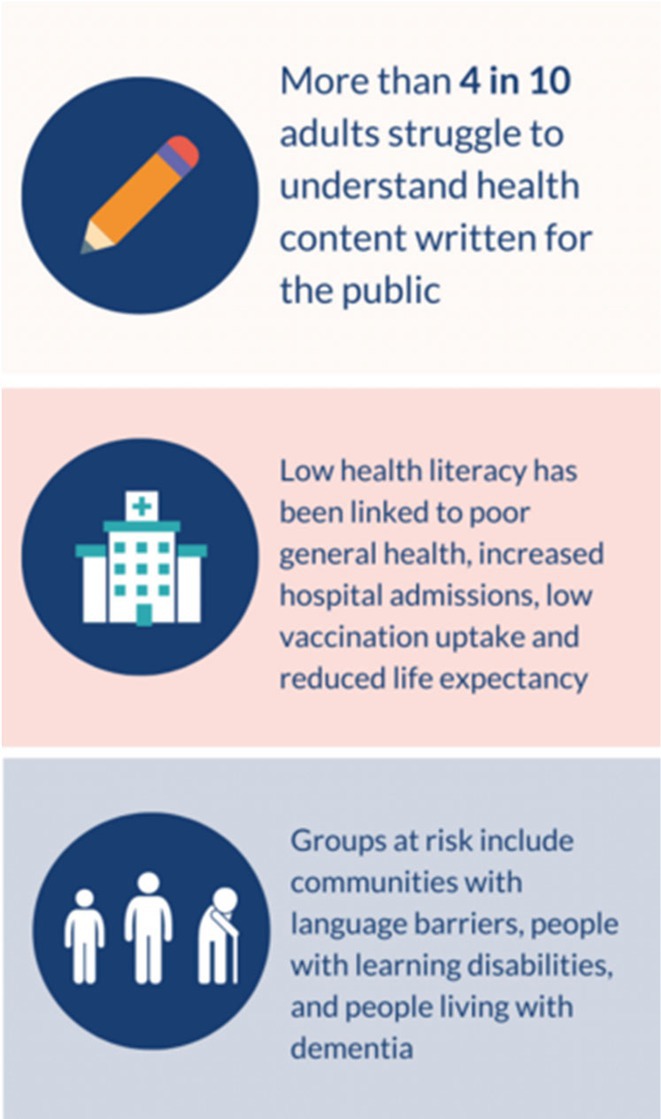

Our research has found that families who communicate openly at home are more likely to engage in discussions and decision‐making with health professionals (Coyne et al. 2017). In a study on health literacy, less than half of participants said they understood their medical diagnosis or treatment, often seeking help from family or the internet (Coyne et al. 2022). This aligns with global findings that four in 10 adults struggle to comprehend health content (World Health Organization 2023).

## 2 Health Literacy Screening

Research highlights the link between health literacy and social determinants of health; for example, socioeconomic status, social behaviours, environmental factors and language spoken at home all influence communication. It is crucial not to make assumptions based on social determinants. However, health professionals rarely use formal tools to assess patient literacy (Hogan et al. 2024). Health literacy assessments could help tailor information more effectively, rather than relying on estimations or providing generic information. Tools such as the single‐item screener ‘How often do you need someone to help you when reading instructions, pamphlets, or other written material?’—have been shown to effectively assess health literacy (Morris et al. 2006). Other tools like the Brief Health Literacy Screener (Chew et al. 2004) and the Newest Vital Sign (Ciccarelli et al. 2010) are also useful for assessing patient understanding.

## 3 Health Literacy and Avoidable Readmission

The link between understanding health information and being able to action this information is important to maintain health outcomes. When patients are discharged, they need to be able to manage their health at home; they need to fully understand their diagnosis and treatment. When patients and family struggle with self‐management and following discharge information, this leads to avoidable hospital readmissions. Avoidable readmissions occur when a patient's unplanned admission to the hospital is related to the initial admission (Australian Commission on Safety and Quality in Health Care 2024).

Improving communication between health professionals, patients and families will enhance both short‐ and long‐term health outcomes (Mileski et al. 2022). Effective communication tailored to the family's needs can improve their management of health conditions at home. Focusing on the family as the unit of care allows for a strengths‐based approach, considering how the family manage their own health and the associated information (Vasegaard et al. 2025). Identifying the family's communication style is key to ensuring the right family members receive and understand the information.

## 4 Recommendations

Take the time to sit down with the family, ask what they want to know, and provide information in a format that suits them. Offering information through oral, written and video formats accommodates different learning styles. Identifying teachable moments during the patient's time with health professionals can also enhance their understanding of the disease (Karvinen et al. 2015). Using videos with closed captions and simplified information can help non‐native speaking families revisit the material at home, improving their understanding (Coyne et al. 2022).

Encouraging family discussions helps to identify unmet needs. This creates opportunities for teach‐back communication, where patients and families repeat what they know to confirm understanding. Nurse‐led teach‐back education has been shown to reduce avoidable hospital readmissions by 30% in patients with cardiovascular disease (Oh et al. 2023). Follow‐up communication both during the hospital stay and after discharge is critical for improving understanding and health outcomes.

In conclusion, reflecting on our communication styles, educating future health professionals and integrating health literacy assessments into care can significantly enhance patient and family health information understanding. By tailoring information and focusing on what the family understands, rather than delivering standard education, we can improve health outcomes and enable improved management of care at home and reduce avoidable readmissions.

## Conflicts of Interest

The authors declare no conflicts of interest.

## Data Availability

The authors have nothing report.
